# Interspecific, Spatial and Temporal Variability of Self-Recruitment in Anemonefishes

**DOI:** 10.1371/journal.pone.0090648

**Published:** 2014-02-28

**Authors:** Hawis H. Madduppa, Janne Timm, Marc Kochzius

**Affiliations:** 1 Marine Science and Technology, Faculty of Fisheries and Marine Science, Bogor Agricultural University (IPB), Bogor, Indonesia; 2 Biotechnology and Molecular Genetics, University of Bremen, Bremen, Germany; 3 Marine Biology, Vrije Universiteit Brussel, Brussels, Belgium; Biodiversity Insitute of Ontario - University of Guelph, Canada

## Abstract

Polymorphic microsatellite DNA parentage analysis was used to investigate the spatio-temporal variability of self-recruitment in populations of two anemonefishes: *Amphiprion ocellaris* and *A. perideraion*. Tissue samples of *A. ocellaris* (*n* = 364) and *A. perideraion* (*n* = 105) were collected from fringing reefs around two small islands (Barrang Lompo and Samalona) in Spermonde Archipelago, Indonesia. Specimens were genotyped based on seven microsatellite loci for *A. ocellaris* and five microsatellite loci for *A. perideraion*, and parentage assignment as well as site fidelity were calculated. Both species showed high levels of self-recruitment: 65.2% of juvenile *A. ocellaris* in Samalona were the progeny of parents from the same island, while on Barrang Lompo 47.4% of *A. ocellaris* and 46.9% of *A. perideraion* juveniles had parents from that island. Self-recruitment of *A. ocellaris* in Barrang Lompo varied from 44% to 52% between the two sampling periods. The site fidelity of *A. ocellaris* juveniles that returned to their reef site in Barang Lompo was up to 44%, while for *A. perideraion* up to 19%. In Samalona, the percentage of juveniles that returned to their natal reef site ranged from 8% to 11%. Exchange of progeny between the two study islands, located 7.5 km apart, was also detected via parentage assignments. The larger Samalona adult population of *A. ocellaris* was identified as the parents of 21% of Barrang Lompo juveniles, while the smaller adult population on Barrang Lompo were the parents of only 4% of Samalona juveniles. High self-recruitment and recruitment to nearby island reefs have important implications for management and conservation of anemonefishes. Small MPAs, preferably on every island/reef, should ensure that a part of the population is protected to enable replenishment by the highly localised recruitment behaviour observed in these species.

## Introduction

Self-recruitment is defined as the proportion of larvae returning to and settling in their natal population, whereas population connectivity is the linking of distinct populations by individual dispersal or migration [1]. These two aspects are fundamental for the management and conservation of living marine resources [2], management of highly harvested species [3], understanding the population dynamics of marine organisms [4], and improving the design of marine reserves [5]. Sufficient self-recruitment and connectivity among populations in marine reserves are believed to prevent local extinction that might otherwise occur as a result of anthropogenic disturbances such as fishing pressure [6]. However, directly measuring the degree of self-recruitment and connectivity in populations of marine organisms is challenging due to the large number and small size of the propagules, the time spent in the dispersive pelagic larval stages and the high mortality. Although the pelagic larval duration (PLD), which varies from days to weeks in fish [7], affects dispersal capability [8], dispersal distances are also potentially influenced by oceanographic processes [9], geographic location and flow variability of ocean currents [10], as well as larval behaviour, such as vertical positioning, swimming and olfactory reef-sensing [11]–[14].

Genetic markers that can be used for determining parentage and relatedness offer an indirect method for measuring self-recruitment and connectivity, thus providing important information on population dynamics. These markers are also widely used for addressing wildlife management issues in a variety of organisms [15]–[19]. A commonly used genetic marker are microsatellites, simple repetitive sequences located throughout the eukaryote nuclear genome [20]. Because of their high variability they are useful for fine-scale ecological studies, such as parentage analysis [21]. Parentage analysis uses data from polymorphic microsatellites for relationship reconstruction based on the maximum likelihood method, where juveniles are assigned to the most likely parent from a data set of potential parents [22]. This method has been proven a powerful tool for investigating self-recruitment in marine fishes [23]–[26], identifying connectivity among fish populations [27]–[28], and determining whether larvae of marine organisms remain close to their origin over small scales (e.g., among groups within a population) [29].

In this study microsatellites are used to study self-recruitment in two species of anemonefish. Spatial patterns of recruitment in anemonefishes are interesting in part due to their unusual symbiosis with anemones, social structure and breeding biology, but also critically important due to the high level of exploitation of these species and their host anemones by the global ornamental fish trade [30]. Anemonefishes have two very different phases in their lifecycle: sedentary adults live in close association with host anemones, while larvae are planktonic. Metamorphosing juveniles recruit to a species-specific host anemone, usually joining a mixed-age group of conspecifics. Within that group, the largest individual is the reproductive female, the second largest the reproductive male, while the remaining individuals are non-reproductive subadults and juveniles [31] [32].

This study focuses on two species of anemonefish: *Amphiprion ocellaris* and *A. perideraion*. With an estimated 145,000 individuals collected from the wild during 1997–2002, *A. ocellaris* is the most frequently traded marine ornamental fish in the global market [30]. The research was conducted in Spermonde Archipelago (Indonesia), where anemonefishes, especially *A. ocellaris*, are intensely collected and overexploitation is indicated [33]. A recent study showed limited connectivity of *A. ocellaris* populations across Indonesia and among shelf areas in Spermonde Archipelago, predicting high self-recruitment in the mid-shelf area of the archipelago [34]. In this study polymorphic microsatellite DNA parentage analysis was used to investigate the degree of self-recruitment, site fidelity, and genetic relatedness of *A. ocellaris* and *A. perideraion* populations of two small islands in Spermonde Archipelago. Understanding the degree of self-recruitment in these populations and their connectivity to neighbouring populations could directly support the design and implementation of effective Marine Protected Area (MPA) networks, as well as the sustainable management and conservation of these species.

## Materials and Methods

### Study Species

*Amphiprion ocellaris* (false clown anemonefish) lives in symbiosis with three anemone species (*Heteractis magnifica, Stycodactyla gigantea*, and *S. mertensii*) and has a planktonic larval duration (PLD) of 8–12 days [32]. It inhabits outer reef slopes or sheltered lagoons to a maximal depth of 15 m. *Amphiprion perideraion* (pink anemonefish) can be associated with four different anemones (*H. magnifica, H. crispa, Mactodactyla doreenis*, and *S. gigantea*; [32]) and has a somewhat longer PLD of 18 days [7]. It typically inhabits lagoon and seaward reefs.

### Study Areas

**Figure 1:**
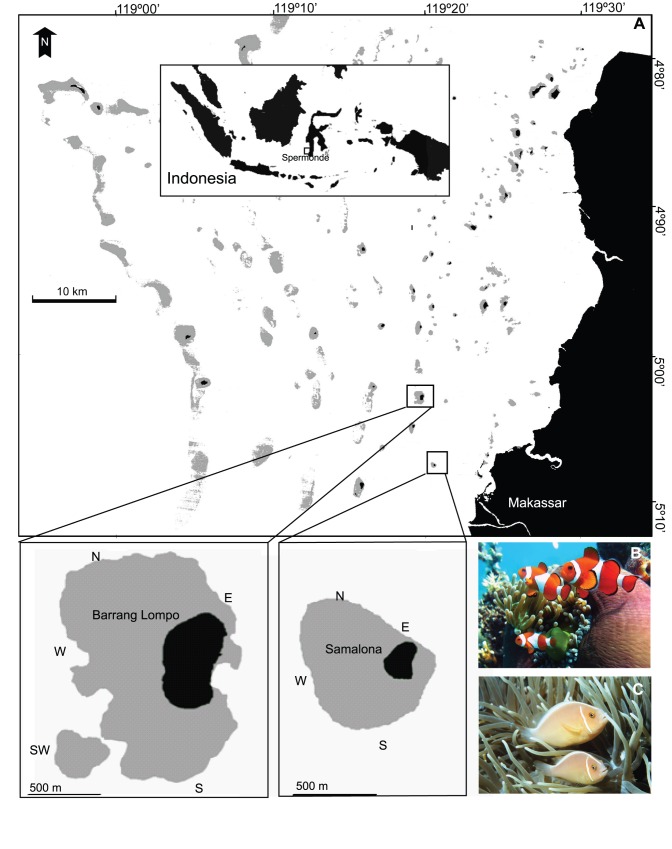
Map of study sites (A): Barrang Lompo and Samalona in Spermonde Archipelago, Indonesia. Barrang Lompo, divided into 5 sample sites. Samalona, divided into 4 sample sites. N: North, E: East, W: West, SW: Southwest, and S: South. Black areas on maps depict land and grey areas shallow coral reefs. (B) A group of *A. ocellaris* (photograph: H. Madduppa); and (C) a pair of *A. perideraion* (photograph: M. Kochzius) in their respective host anemones.

Spermonde Archipelago (South Sulawesi, Indonesia) (Fig. 1) comprises about 150 islands [35] and is situated at the southwestern tip of Sulawesi in the centre of marine biodiversity, the so-called “Coral Triangle”. This archipelago is affected by the very strong Indonesian Throughflow (ITF) current, which connects the Pacific Ocean with the Indian Ocean [36]. This setting potentially enhances the dispersal of marine organisms in Spermonde Archipelago, though interactions between oceanographic processes and larval behaviour may enable larvae to stay close to their natal population [37]. About 50,000 people live in Spermonde Archipelago and coral reef resources form an important part of their livelihoods. Therefore, these reefs are under threat from a variety of anthropogenic activities, including destructive fishing practices and land-based pollution [38]. The present study was conducted at two small islands, Barrang Lompo and Samalona (Fig. 1), located in the mid-shelf region of Spermonde Archipelago. Barrang Lompo (5°02′52.07″S, 119°19′45.25″E), located 13 km west of Makassar, is 19 ha in size and inhabited by about 5,000 people. Its fringing coral reefs have been impacted by dynamite-fishing and local sewage pollution [39]. Samalona (5°07′30.48″S, 119°20′36.48″E), located 5 km west of Makassar, is 2 ha in size, and inhabited by about 80 people. Samalona has been developed by local people for small-scale tourism and is therefore relatively protected from destructive fishing activities. However, Samalona′s reefs have been impacted by anchor damage and pollution from Makassar [39]. On both study islands, the fringing reefs extend from the shore to depths of 2–10 m, where the substrate changes to soft sediment. The outer circumferences of the fringing reefs are 2.5 km and 1.48 km at Barrang Lompo and Samalona, respectively.

### Field Sampling Methods

In order to completely sample the populations of *A. ocellaris* and *A. perideraion* on Barrang Lompo and Samalona, scuba divers systematically searched the entire area of the fringing reefs for host anemones. To facilitate this process and provide more information on location of the anemonefishes, the reef in Barrang Lompo was subdivided into five sites and the reef in Samalona into four. At each site, all host anemones were located and associated anemonefishes identified and counted. To obtain tissue for genetic analysis of the anemonefishes, two small aquarium nets were used to carefully capture each individual fish and a small fin clip of the caudal fin was collected. The length of the fish was measured and then it was immediately released back to the host anemone. Fin-clipped individuals could be readily identified, so resampling was not a problem and it was possible to visually ensure that all individuals associated with a particular anemone were sampled. Each tissue sample was put into a separate tube and all associated data (fish species, size, date, location, and anemone species) was recorded immediately. Tissue samples were preserved in 96% ethanol after the dive and stored at 4°C in the laboratory until DNA extraction.

The sex and reproductive status of individuals within each group on a host anemone were determined by body size. The largest fish was assumed to be the reproductive female, the second largest the reproductive male, and all others were assumed to be non-breeding individuals [31] [40]. Non-breeding individuals will be referred to as “juveniles” henceforth.

A total of 364 tissue samples of *A. ocellaris* and 105 tissue samples of *A. perideraion* were collected at the two islands (Table 1). In Barrang Lompo, 88 *A. ocellaris* individuals were sampled from 17 anemones in October 2008 and May 2009. In Samalona, a total of 276 individuals were sampled from 83 anemones in May 2009. For *A. perideraion*, 105 individuals were sampled from 35 anemones in Barrang Lompo in May 2009.

**Table 1:** Sample collection in Spermonde Archipelago, Indonesia.

Site	Geographic coordinates	Length of reef	*No. of Anemone* *	*A. ocellaris*			*A. perideraion*	
		surveyed (m)		*Adults*	Juveniles (2008)	Juveniles (2009)**	*Adults*	Juveniles (2009)
Barrang Lompo								
West	S 05° 02.541′ E 119° 19.355′	763	3	3	7	8	6	16
Southwest	S 05° 03.324′ E 119° 19.276′	293	2(1)	2	2	6	25	36
North	S 05° 02.507′ E 119° 19.571′	677	8(2)	14	10	5	1	0
South	S 05° 03.317′ E 119° 19.490′	395	5	7	5	10	9	6
East	S 05° 03.280′ E 119° 19.836′	383	2	4	2	3	0	6
Total				30	26	32	41	64
Samalona								
West	S 05° 07.010′ E 119° 20.006′	220	17	34		18		
North	S 05° 07.009′ E 119° 20.007′	560	22	44		28		
East	S 05° 07.005′ E 119° 20.009′	470	30	58		53		
South	S 05° 07.011′ E 119° 20.009′	230	14	28		13		
Total				164		112		

*Parentheses: the number of anemones with a single resident anemonefish and thus excluded from the relatedness analysis.

**juveniles collected in the 2009 at Barrang Lompo for *A. ocellaris* were limited to size a maximum of 2 cm total length.

### Ethics Statement

Fin-clipping is a non-destructive, minimally invasive and the most commonly used method to obtain tissue from living fishes in the wild (e.g. [24] [26] [28]) and in aquaculture (e.g. [41]). We took great care to minimise harm, and ensure survival by safely releasing the fishes back into their host anemones. Tissue sampling of these anemonefishes was permitted within the framework of the German-Indonesian SPICE project (Science for the Protection of Indonesian Coastal Ecosystems), in cooperation with the Hasanuddin University, Makassar, Indonesia.

### DNA Extraction, Microsatellite Amplification and Allele Sizing

Genomic DNA from *Amphiprion ocellaris* and *A. perideraion* was extracted with the NucleoSpin tissue extraction kit (Macherey-Nagel), following the manufacturer’s guidelines. All DNA extracts were analysed by gel electrophoresis to monitor DNA quality prior to polymerase chain reaction (PCR) amplification of microsatellite loci. DNA extracts were stored at −20°C.

PCRs were carried out in a total volume of 25 µl, containing 2.5 µl 10x PCR buffer, 3 µl 25 mM MgCl_2_, 1 µl 2 mM each dNTP, 1 µl each 10 mM primer forward and reverse, 0.1 µl (5 unit/µl) Taq polymerase (F100L Taq DNA), 1 µl (1–10 ng) genomic DNA. PCRs were performed in a TProfessional Thermocycler (Biometra) or a Mastercycler ep (Eppendorf) with the following thermo-profile: 94°C for 2 minutes, followed by 35 cycles of 94°C for 30 seconds as the denaturing step, 50–65°C for 30 seconds as the annealing step (the optimal annealing temperature varies between primers, see Table 2), 72°C for 1 minute for the polymerisation, and finally 72°C for 2 minutes.

**Table 2:** Polymorphic microsatellite loci used as genetic markers for *Amphiprion ocellaris* and *A. perideraion*.

Locus	Repeat motif	Ann.Ao	Ann.Ap	Primer sequences (5′-3′)	*Dye*	Primer Source	Reference
Cf 9	Tetranucleotide	60	–	F: CTC TAT GAA GAT TTT T	HEX	*Amphiprion percula*	Buston et al. 2007
				R: GTA CAT GTG TTT CCTC			
Cf 42	Ditetranucleotide	55	53	F: AAG CTC CGG TAA CTC AAA ACT AAT	HEX	*A. percula*	Buston et al. 2007
				R: GTC ATC TGA TCC ATG TTG ATG TG			
Cf 29	Dinucleotide	58	–	F: TTC TTT ATC CCC TTG TTT ATT TCT AA	FAM	*A. percula*	Buston et al. 2007
				R: AAG CCT CCT TTC CAA AAC CAC TCA			
45	Dinucleotide	62	–	F: TCA ACT GAA TGG AGT CCA TCT GG	FAM	*A. polymnus*	Quenouille et al. 2004
				R: CCG CCG CTA GCC GTG ACA TGC AA			
120	Dinucleotide	62	68	F: TCG ATG ACA TAA CAC GAC GCA GT	HEX	*A. polymnus*	Quenouille et al. 2004
				R: GAC GGC CTC GAT CTG CAA GCT GA			
AC1578	Dinucleotide	53	55	F: CAG CTC TGT GTG TGT TTA ATG C	FAM	*A. clarkii*	Liu et al. 2007
				R: CAC CCA GCC ACC ATA TTA AC			
AC137	Dinucleotide	58	55	F: GGT TGT TTA GGC CAT GTG GT	FAM	*A. clarkii*	Liu et al. 2007
				R: TTG AGA CAC ACT GGC TCC T			
AC915	Dinucleotide	–	58	F: TTG CTT TGG TGG AAC ATT TGC	HEX	*A. clarkii*	Liu et al. 2007
				R: TCT GCC ATT TCC TTT GTT C			

[Abbreviations: Ann. = Annealing temperature; Ao = *A. ocellaris*; A.p = *A. perideraion*; Dye = fluorescence dye].

Twenty microsatellite loci were amplified, using primers from other *Amphiprion* species [29] [42] [43]. Of these, seven were polymorphic in *A. ocellaris* and five in *A. perideraion*. These loci (Table 2) were amplified by PCR with a labelled forward primer containing a 5′-fluorescent dye (FAM or HEX). PCR products were diluted in pure water prior to fragment analysis. Dilution factors were determined empirically for each locus, and ranged from 1∶5 to 1∶30. For fragment analysis, 1 µl of diluted PCR product was combined with 8.85 µl HiDi™ formamide and 0.15 µl GENESCAN LIZ-500 size standard (Applied Biosystems). Microsatellite fragments were size fractioned using an ABI 3730 48 capillary sequencer with a capillary of 50 cm length (Applied Biosystems). Allele sizes were determined and corrected with PEAK SCANNER v1.0 (Applied Biosystems) and GENEMARKER v1.85 (SoftGenetics GeneMarker). The program MICROCHECKER was used to detect null alleles and to identify irregularities in the data, including mistyped allele sizes, typographic mistakes, as well as scoring errors [44].

### Summary Statistics, Test of Hardy-Weinberg Equilibrium, and Linkage Disequilibrium

The total number of alleles per locus, allele frequencies, observed and expected heterozygosities [45], and the Polymorphic Information Content (PIC) of each locus [46] were calculated with the program CERVUS 3.0 [47]. The levels of polymorphism at each microsatellite locus was ranked as: (1) highly informative (PIC >0.5), (2) reasonably informative (0.5≥PIC ≥0.25), or (3) slightly informative (PIC <0.25), following Botstein et al. [46]. PIC values are determined based on the frequency of alleles at a given locus. Hardy-Weinberg equilibrium (HWE) exact tests and loci combinations for linkage disequilibrium with the Markov chain methods were conducted using GENEPOP on the web [48] [49]. In order to test the null hypothesis of HWE, the probability test was conducted and the alternative hypothesis of heterozygote deficiency was tested. The null hypothesis of linkage disequilibrium for the diploid case was tested through pairwise comparisons of loci. For all Markov chain methods, parameters used were the default settings for dememorisation number (1000), number of batches (100), and iterations per batch (1000). Significance levels were adjusted with sequential Bonferroni corrections for multiple tests with *P*≤0.05. The coefficient of inbreeding (*F*_IS_) was calculated with the program FSTAT 2.9.3 [50] in order to detect non-random mating within populations [51]. The *F*_IS_-value ranges from −1 (extreme outbreeding), 0 (no inbreeding), to +1 (complete inbreeding). The software CONVERT 1.3.1 [52] was used to obtain the correct file formats for the various programs applied.

### Parentage Analysis

Microsatellite DNA parentage analysis was conducted with FAMOZ [53]. Using a likelihood-based approach [54], juveniles were assigned to a single parent or parent pair in order to select the most likely parent from a pool of potential parents [22]. Suitability of this program for such analyses in fish populations was shown in several studies [23] [26] [27]. In this program, the exclusion probability [55] is generated, which uses incompatibilities between parents and offspring to reject particular parent-offspring hypotheses [22]. The log-likelihood ratios or logarithm of odds (LOD) scores were calculated with this program for each parent/offspring association, using microsatellite allele frequencies calculated by CERVUS 3.0. LOD score threshold values for each error type were taken from the intersection of offspring with genotyped parents and offspring generated according to allele frequencies. Simulations have to be performed to determine suitable thresholds, error levels, and the impact of scoring errors for each population [56]. Therefore, five different error rates were evaluated for all data sets (Table S1). Each error rate was evaluated with 10,000 replicates of simulated offspring. Finally, to compensate for error in scoring parents or offspring genotypes, the presence of null alleles, and marker mutation [47], an error rate of 0.01 was chosen for parentage analysis for all populations of *A. ocellaris*, and an error rate of 0.001 for *A. perideraion* populations (Table S1). LOD score threshold values for *A. ocellaris* for single parent and parent pair were 1.9 and 5.9 for the Barrang Lompo population, respectively. In the population from Samalona, the LOD score threshold values were 2.5 and 6.7, respectively. For *A. perideraion*, LOD score threshold values for single parent and parent pair were 2.8 and 7.9, respectively. Unassigned juveniles of *A. ocellaris* to a single parent or parent pair in Barrang Lompo were assigned to potential parents in Samalona, and vice versa. These two data sets were also simulated to obtain suitable LOD thresholds (Table S1). All tests showed high cumulative exclusion probabilities (>0.9). The parameters for the parent/offspring assignment decision were as follows [57]: (1) individuals were assigned to the most likely single parent if the LOD score was equal or larger than the single parent threshold and (2) individuals were assigned to the most likely parent pair if the LOD score was equal or larger than both the single parent and parent pair threshold. No parent assignment was made if the LOD score was less than the single parent threshold.

### Dispersal Distances and Spatial Patterns of Recruitment

The results of parentage analysis were used to calculate the proportions of juveniles that: (1) recruited to the same anemone inhabited by their parents; (2) recruited to the natal site; (3) recruited to an adjacent site on their natal island reefs; (4) recruited to a non-adjacent site on their natal island reefs; (5) recruited from the other study island, or (6) had no parent identified from either study island. Satellite images were used to measure distances among sites both within and between islands. This information was used to estimate a minimum dispersal distance based on the locations of parent and offspring.

### Genetic Relatedness

In addition to parentage analysis, a genetic relatedness index was calculated to determine whether individuals sharing an anemone were related to one another. Genetic relatedness among the individuals inhabiting each anemone (“anemone group”) was conducted using KINGROUP v2 [58]. In this program, the method “kinship pairwise” [59] was chosen to construct the coefficient of relatedness (*r*), which estimates patterns of kinship in natural populations. An *r* value less than zero means that individuals from the same anemone are unrelated, and an *r* value greater than zero means that individuals within anemones are related. Relatedness in anemone groups was calculated for three populations: *A. ocellaris* from Barrang Lompo (n = 53; 17 groups), *A. ocellaris* from Samalona (n = 276; 83 groups), and *A. perideraion* from Barrang Lompo (n = 100; 35 groups). Allele frequencies from each of the three populations were estimated separately to calculate relatedness. A permutation test was used to compare the relatedness values obtained between two individuals from the same anemone-group and individuals within the same island. The statistical analysis was conducted in BASE SAS 9.3 [60]. The mean coefficient of relatedness within reef-sites and within island were also calculated for both species.

## Results

### Summary Statistics, Hardy-Weinberg Equilibrium, and Linkage Disequilibrium

All markers used in the analysis were ranked as highly informative (Table 3). The average PIC value was 0.766±0.161 (mean ± SD) and ranged from 0.465 (locus 120) to 0.925 (locus Cf42) in the *Amphiprion ocellaris* populations, and 0.799±0.154 (mean ± SD) with a range of 0.601 (locus AC915) to 0.969 (locus Cf42) in the *A. perideraion* population. The number of alleles varied between five (locus 120) and 30 (loci Cf42 and AC137) in the *A. ocellaris* populations, and between five (loci AC915 and AC1578) and 57 (Cf42) in the *A. perideraion* population. The observed heterozygosity (*H_o_*) ranged from 0.5 (120) to 0.977 (Cf29), and the expected heterozygosity (*H_e_*) ranged from 0.539 (120) to 0.931 (Cf42) in the *A. ocellaris* populations. In the *A. perideraion* population, *H_o_* ranged from 0.267 (AC1578) to 0.952 (Cf42), and *H_e_* ranged from 0.653 (AC915) to 0.975 (Cf42). The probability test indicated that locus AC1578 was not in HWE (*P*<0.01; Table 4). However, the alternative hypothesis of heterozygote deficiency was rejected (*P* = 0.087). Therefore, this locus remained in the dataset for further analysis. In the *A. perideraion* population, HWE tests indicated two loci (Cf42 and AC1578) deviating from equilibrium. Only locus 45 showed evidence of null alleles. However, these loci remained in the dataset for further analysis as well. No significant linkage disequilibrium was found for any loci pair, indicating that all loci could be considered as independent.

**Table 3:** Summary statistics for *Amphiprion ocellaris* (two populations) over seven polymorphic microsatellite loci, and *A. perideraion* over five polymorphic microsatellite loci.

Locus	Allele (bp)	*k*	*PIC*	*F_IS_*	*H_o_*	*H_e_*	Prob.	H_1_
*Amphiprion ocellaris:* Barrang Lompo population (n = 88)								
Cf9	262–298	10	0.765	−0.012	0.807	0.797	0.869	0.519
Cf29	190–234	18	0.905	−0.065	0.977	0.917	0.879	0.95
Cf42	262–320	25	0.920	−0.027	0.955	0.930	0.329	0.432
45	216–246	12	0.648	0.225	0.523	0.674	<0.01	0.013
120	454–462	5	0.465	0.073	0.500	0.539	0.265	0.076
AC137	256–322	20	0.912	0.003	0.920	0.923	0.679	0.192
AC1578	250–264	8	0.755	0.082	0.727	0.792	0.117	<0.001
mean				0.039				
*Amphiprion ocellaris:* Samalona population (n = 276)								
Cf9	262–302	11	0.772	−0.019	0.815	0.800	0.861	0.469
Cf29	200–248	21	0.890	−0.075	0.967	0.900	0.004	1
Cf42	258–324	30	0.925	−0.004	0.935	0.931	0.705	0.597
45	216–246	14	0.551	0.013	0.572	0.580	0.081	0.323
120	450–470	9	0.523	−0.004	0.594	0.592	0.91	0.599
AC137	250–328	30	0.920	0.015	0.913	0.926	0.334	0.031
AC1578	250–266	9	0.781	0.033	0.783	0.810	<0.001	0.087
mean				−0.006				
*Amphiprion perideraion:* Barrang Lompo population (n = 105)								
Cf42	258–408	57	0.969	0.023	0.952	0.975	<0.001	<0.001
120	456–480	11	0.787	0.052	0.771	0.813	0.759	0.316
AC137	276–336	24	0.925	0.154	0.790	0.934	0.014	<0.001
AC915	218–230	5	0.601	0.052	0.619	0.653	0.808	0.265
AC1578	250–258	5	0.634	0.617	0.267	0.695	<0.001	<0.001
mean				0.179				

For each locus, the data given are allele size range (bp = base pairs), number of alleles (*k*), polymorphic information content (*PIC*), the coefficient of inbreeding (*F_IS_*), the observed (*Ho*) and expected (*He*) heterozygosity, and *P* values for Hardy-Weinberg exact test (Prob. = Probability test; H_1_ = Heterozygote deficiency).

**Table 4:** Recruitment within-island (self-recruitment) and between-island of anemonefishes in Barrang Lompo and Samalona, (Spermonde Archipelago. Indonesia).

Sampling Date	Population		Within-Island Parentage			Between-Island Parentage		
	Adults	Juveniles*	Single-parent assignment	Parent-pair assignment	Total Self-Recruitment	Single-parent assignment	Parent-pair assignment	Total Between-Island Recruitment
**A. ** ***Amphiprion ocellaris***								
**Barrang Lompo: 17 host anemones**								
Oct-Nov 2008	31	25	12 (48%)	1 (4%)	13 (52%)	6 (24%)	1 (4%)	7 (28%)
May 2009		32	14 (43.8%)	0 (0%)	14 (43.8%)	6 (19.4%)	0 (0%)	6 (19.4%)
TOTAL	31	57	26 (45.6%)	1 (0.02%)	27 (47.4%)	12 (21.1%)	1 (1.8%)	13 (22.8%)
**Samalona: 83 host anemones**								
May 2009	164	112	68 (60.7%)	5 (4.5%)	73 (65.2%)	4 (3.6%)	0 (0%)	4 (3.6%)
**B. ** ***Amphiprion perideraion***								
**Barrang Lompo: 35 host anemones**								
May 2009	41	64	28 (43.8%)	2 (3.1%)	30 (46.9%)			

*The term juveniles as used here includes all non-reproductive individuals.

### Parentage Analyses and Spatial Patterns of Recruitment

**Figure 2:**
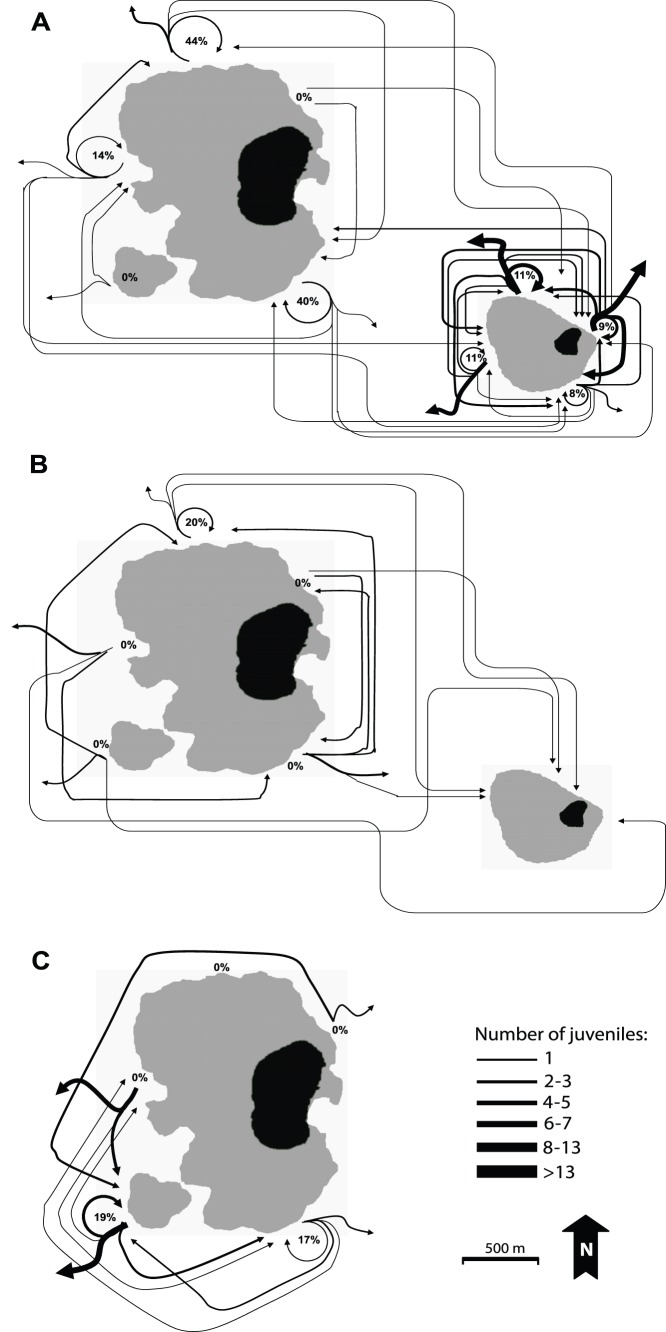
The spatial patterns of recruitment (juveniles movement) of *Amphiprion ocellaris* at (A) Barrang Lompo in 2008 and *A. ocellaris* at Samalona in 2009; (B) *A. ocellaris* in 2009; as well as between two islands. The two islands are not oriented to each other as shown here, see Fig. 1 for detail; and (C) *A. perideraion* at Barrang Lompo in 2009. The percentage of site fidelity of *A. ocellaris* (2008 in Barrang Lompo, 2009 in Samalona) and *A. perideraion* (2009) for each reef site is also shown. All juveniles in the analysis were identified as being the progenies of adults of the present study sites using DNA parentage analysis with the program FAMOZ. Black area: island; grey area: shallow coral reef.

Single-parent or parent-pair assignments were made for 94 of the 169 *Amphiprion ocellaris* juveniles and 28 of the 64 *A. perideraion* juveniles (Table 4). Of these, the majority represented within-island recruitment (44–65%), with between-island recruitment quite low for *A. ocellaris* on Samalona (4%) and higher on Barrang Lompo (23%). Within-island recruitment patterns showed that juveniles were most likely to be found on reef-sites that were adjacent to their natal site and least likely to be found on non-adjacent sites (Fig. 2).

### Site Fidelity

Juveniles of *A. ocellaris* and *A. perideraion* were staying at their natal site in different proportions (Fig. 2). The percentage of juveniles of *A. ocellaris* that returned to their natal site in Barang Lompo range from 0 to 44%, while *A. perideraion* ranged from 0 to 19%. In the Samalona, the percentage of *A. ocellaris* juveniles that returned to their origin site ranged from 8% to 11%. However, most of them settled and dispersed close to their natal site within their island.

### Genetic Relatedness

**Figure 3:**
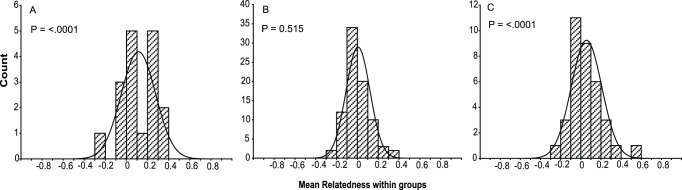
Distribution of the average relatedness (*r*) within group anemones and comparison with the mean relatedness within the island (Table 5). (A) *Amphiprion ocellaris* in Barrang Lompo (53 individuals, 17 groups; *P* = <.0001), (B) *A. ocellaris* in Samalona (276 individuals, 83 groups; *P* = 0.515), and (C) *A. perideraion* in Barrang Lompo (100 individuals, 35 groups; *P* = <.0001).

The mean coefficient of relatedness of *Amphiprion ocellaris* individuals within anemone groups at Barrang Lompo was 0.108±0.162 (mean ± SD; *n* = 17 groups), which was significantly higher than the mean within-island relatedness of 0.047±0.111 (SD) (*P* = <.0001, Fig. 3A, Table 5). Thus, at Barrang Lompo, *A. ocellaris* sharing an anemone were more closely related to one another than to other individuals on the island. In contrast, at Samalona, the mean coefficient of relatedness of *A. ocellaris* individuals within anemone groups was 0.001±0.114 (mean ± SD; *n* = 83 groups), not significantly different than the mean of 0.004±0.056 (SD) for within-island relatedness (*P* = 0.515, Fig. 3B). The value for within-anemone group relatedness of *A. perideraion* at Barrang Lompo was 0.051±0.151 (mean ± SD; *n* = 35 groups) significantly higher than the mean of 0.008±0.164 (SD) within-island relatedness (*P* = <.0001, Fig. 3C, Table 5). Within-reef relatedness ranged from −0.04±0.24 to 0.30±0.01 (mean ± SD) for *Amphiprion ocellaris* populations, while for the *A. perideraion* population it ranged from −0.06±0.15 to 0.15±0.12 (mean ± SD).

**Table 5:** The coefficient of relatedness (*r*) within reef-sites and within island at Barrang Lompo and Samalona for *Amphiprion ocellaris* and *A. perideraion*.

Population	Barrang Lompo				Samalona	
	*Amphiprion ocellaris*		*Amphiprion perideraion*		*Amphiprion ocellaris*	
	*r* (mean)	±SD	*r* (mean)	±SD	*r* (mean)	±SD
**Within reef-sites**						
West	0.30	0.01	0.11	0.15	0.10	0.23
North	0.09	0.20	–	–	0.15	0.22
South	0.02	0.07	0.15	0.12	0.04	0.21
East	0.01	0.01	−0.05	0.09	−0.04	0.24
Southwest	0.27	0.00	−0.06	0.15		
**Within-island**	0.047	0.111	0.008	0.164	0.004	0.056

## Discussion

### Self Recruitment

The present study revealed high self-recruitment of anemonefishes within reefs surrounding small islands, with 47–65% of *Amphiprion ocellaris* and *A. perideraion* progeny staying on their natal island. Self-recruitment of *A. ocellaris* (65%) at Samalona is higher than the 42% reported from a previous study on the sibling species *A. percula* [26]. The high self-recruitment in Samalona and Barrang Lompo is in agreement with evidence of restricted gene flow revealed in Spermonde Archipelago and across the Indo-Malay Archipelago [34]. Restricted dispersal might be triggered by the sheltered environment within the mid-shelf of Spermonde Archipelago, where the study islands are located, compared to outer shelf of the archipelago, which are strongly affected by the Northwest Monsoon [35].

Self-recruitment rates in Barrang Lompo (*Amphiprion ocellaris*: 47.4%; *A. perideraion*: 46.9%) were lower than in Samalona (*A. ocellaris*: 65.2%). Self-recruitment in *Amphiprion ocellaris* varied from 44% to 52% between the two sampling periods in Barrang Lompo. One possible hypothesis is that the self-recruitment rate on Barrang Lompo may be biased by high fishing pressure [38] [61] [62]. Parent anemonefish could be removed by ornamental fishermen, thus deflating the estimate of self-recruitment.

The pelagic larval duration (PLD) varies from days to weeks in different species of coral reef fish [7] and thus may influence their dispersal distance. Due to pelagic dispersal of eggs and larvae, most marine species have been considered as open populations [63], even though this is under discussion [64] [65]. In an open marine population, fish larvae are assumed to be transported by predominant currents during their pelagic stage over long distances, facilitating high connectivity among populations [3]. However, many recent studies using different methodologies and molecular markers estimated a high self-recruitment level in different marine fish species with different PLDs, suggesting low dispersal leading to low connectivity among populations. Examples are an assignment test using microsatellite loci in *Tripterygion delaisi* (PLD: 16–21 days, self-recruitment (SR): 66%; [66]), otolith marking in *Pomacentrus amboinensis* (PLD: 16–19 days, SR: 15%; [67]), tetracycline mass-marking and parentage analysis in *Amphiprion polymnus* (PLD: 9–12 days, SR: 16–32%; [23]), and otolith microstructure and microchemistry analysis in *Sebastes melanopus* (PLD: 83–174 days, SR: 66–87%; [68]). The findings mentioned above are supported by the current study. Therefore, it seems as if the dispersal of pelagic fish larvae may be more restricted and marine systems may not be as open as previously assumed.

### Site Fidelity and Spatio-temporal Patterns of Recruitment

Larval dispersal in marine organisms can vary from less than 1 km to 100 s km [27] [28] [69]–[71], which may affect their spatial and temporal recruitment. This study shows that most anemonefish larvae settle less than 2 km from their natal reef site. The populations of Barrang Lompo and Samalona were connected by a moderate exchange rate of 10 to 19%. A study on self-recruitment in *Amphiprion polymnus* showed that even though no individuals settle into the same anemone as their parents, most of them settled in other anemones close to them [23], which is in concordance with the findings in *A. ocellaris* and *A. perideraion* of the current study. Recruitment of *A. ocellaris* in Barrang Lompo was high but showed a slight difference between the two sampling periods. This could be natural variation or due to the collection for marine ornamental trade, as explained above. With a proper estimation of connectivity and degree of self-recruitment in marine populations, it might be possible to improve the design of marine reserves. For example, a series of small Marine Protected Areas on each island with short distance to each other can serve to maintain local populations both by self-recruitment and through larval dispersal from nearby reserves [1] [26].

The connectivity within and among populations might be also influenced by the behaviour of the planktonic larvae, as shown in the coral reef fish *Amblyglyphidodon curacao* that has the capability to swim against a current, control its vertical position, and locate a reef [72]. The early inception of the active larval movement is important to mediate the dispersal potential [70], such as olfactory sensing that might also influence larval movement [73]. Planktonic larvae of marine fish are assumed to be able to recognise and return to their natal site. It was shown that *Amphiprion ocellaris* imprints itself to its species-specific host sea anemone using olfactory cues, which are genetically inclined towards olfactory recognition of their host anemone [12]. However, this study found that juveniles come back to their natal reef but not to their natal anemone. In order to facilitate retention, fish larvae may use odour recognition [74]. In addition, the connectivity within and among populations in anemone fish is limited by their relatively short PLD of about 8–12 days. However, exploitation of species, leading to decreased population density and body size [33] [75] [76], could also reduce larval abundance and reduce the dispersal kernel and effective connectivity distances [77].

### Genetic Relatedness

The average genetic relatedness in Barrang Lompo populations showed that *A. ocellaris* and *A. perideraion* individuals within an anemone were more closely related to one another than to other individuals on the island, indicating that fish larvae do not disperse far from their parents. The values of genetic relatedness are in concordance with the coefficient of inbreeding. The current study observed low but positive values of the inbreeding coefficient for *A. ocellaris* and *A. perideraion* at Barrang Lompo, meaning that there is an indication to inbreeding in these populations.

The close relationship between individuals within a site at Barrang Lompo might be explained by several mechanisms. Many marine fishes have the ability to recognise their relatives in order to avoid inbreeding and competition [78]. However, due to a low abundance of anemones as a result of removal by the ornamental fishery [33], the pelagic larvae might not be able to avoid settling to their natal anemone. Close relatedness between individuals in an anemone may lead to inbreeding, which increase homozygosity, causes deleterious alleles in the first generation and reduces the adaptive capacity of species, and could lead to increased mortality [79] [80].

In contrast, individuals inhabiting an anemone were unrelated in *A. ocellaris* at Samalona. These results agree with findings in *A. percula* [29] and *Dascyllus aruanus* [81], forming groups consisting of unrelated individuals.

### Implications for Management and Conservation

Anemonefishes have been exploited for marine ornamental fishery and traded globally for many years. Most marine ornamental fish species are collected from the wild, with Indonesia and the Philippines as the major exporters [30]. In order to avoid overexploitation and to reduce the pressure on natural populations, some efforts have been made to rear ornamental fish species [82]–[84]. Anemone fish such *A. ocellaris* have been successfully bred in captivity [85], which is potentially a good solution to meet the high demand. However, mariculture needs comprehensive knowledge of the biology of the reared fish species and is expensive [83] [86]. Therefore, it seems that mariculture would be difficult to be implemented for many ornamental species, especially in developing countries. In Spermonde Archipelago, *A. ocellaris* is the most collected marine ornamental fish species and this fishery has a negative impact [33]. However, there is no quota for anemonefishes in Indonesia so far.

The two focal species (*A. ocellaris* and *A. perideraion*) have not yet been included in the CITES list (Convention on International Trade in Endangered Species of Wild Fauna and Flora), which aims to prevent overexploitation by controlling international trade [87]. Considering the high level of exploitation, a proper management plan and conservation strategy should be implemented for this species in Spermonde Archipelago [33]. Implementation of MPAs as a tool to manage fisheries and marine biodiversity has been used to protect coral reefs from overexploitation, increase fish populations, restore ecosystem health, and prevent local extinctions [88]–[91]. However, determining the optimal size and location of self-sustaining MPAs is essential for promoting population persistence [6] [92]–[94].

The high amount of self-recruitment of *Amphiprion ocellaris* and *A. perideraion* found in the current study gives valuable information for management and conservation strategies within the region. High levels of self-recruitment imply that the populations are more vulnerable to local fishing activity [95]. While further studies on other marine organisms are needed, the current study suggests that single marine protected areas (MPAs) are not suitable as sources for the replenishment of exploited populations. Small MPAs, preferably at every island or reef, should ensure that a part of the population is protected to allow for replenishment by self-recruitment. In addition, the population size should be estimated in order to establish appropriate catch quotas.

## Supporting Information

Table S1 **Error rates on parentage assignment and gene flow for different populations of parents and juveniles.** Bold numbers indicate the LOD thresholds used for parentage analysis in the program Famoz. [Abbreviations: Juvs = Juveniles; BL = Barrang Lompo; S = Samalona; SP = single parent; PP = parent pair].
(DOC)

## References

[1] Jones G, Almany G, Russ G, Sale P, Steneck R, et al (2009) Larval retention and connectivity among populations of corals and reef fishes: history, advances and challenges. Coral Reefs 28: 307–325.

[2] Fairweather PG (1991) Implications of ‘supply-side’ ecology for environmental assessment and management. Trends Ecol Evol 6: 60–63. 21232426 10.1016/0169-5347(91)90125-H

[3] Roberts CM (1997) Connectivity and management of Caribbean coral reefs. Science 278: 1454–1457. 9367956 10.1126/science.278.5342.1454

[4] Underwood AJ, Fairweather PG (1989) Supply-side ecology and benthic marine assemblages. Trends Ecol Evol 4; 16−20. 10.1016/0169-5347(89)90008-6 21227303

[5] Almany G, Connolly S, Heath D, Hogan J, Jones G, et al (2009) Connectivity, biodiversity conservation and the design of marine reserve networks for coral reefs. Coral Reefs 28: 339–351.

[6] Sala E, Aburto-Oropeza O, Paredes G, Parra I, Barrera JC, et al (2002) A general model for designing networks of marine reserves. Science 298: 1991–1993. 12471258 10.1126/science.1075284

[7] Wellington GM, Victor BC (1989) Planktonic larval duration of one hundred species of Pacific and Atlantic damselfishes (Pomacentridae). Mar Biol 101: 557–567.

[8] Lester SE, Ruttenberg BI (2005) The relationship between pelagic larval duration and range size in tropical reef fishes: a synthetic analysis. Proc Biol Sci 272: 585–591. 16007745 10.1098/rspb.2004.2985 PMC1564084

[9] Wilson DT, Meekan MG (2001) Environmental influences on patterns of larval replenishment in coral reef fishes. Mar Ecol Prog Ser 222: 197–207.

[10] Sponaugle S, Cowen RK, Shanks A, Morgan SG, Leis JM, et al (2002) Predicting self-recruitment in marine populations: Biophysical correlates and mechanisms. Mar Pollut Bull 70: 341–375.

[11] Leis JM, Sweatman HPA, Reader SE (1996) What the pelagic stages of coral reef fishes are doing out in blue water: daytime field observations of larval behavioural capabilities. Mar Freshw Res 47: 401–411.

[12] Arvedlund M, Nielsen LE (1996) Do the anemonefish *Amphiprion ocellaris* (Pisces: Pomacentridae) imprint themselves to their host sea Anemone *Heteractis magnifica* (Anthozoa: Actinidae)? Ethology 102: 197–211.

[13] Fisher R, Bellwood DR, Job S (2000) Development of swimming abilities in reef fish larvae. Mar Ecol Prog Ser 202: 163–173.

[14] Kingsford MJ, Leis JM, Shanks A, Lindeman KC, Morgan SG, et al (2002) Sensory environments, larval abilities and local self-recruitment. Bull Mar Sci 70: 309–340.

[15] Bruford MW, Wayne RK (1993) Microsatellites and their application to population genetic studies. Curr Opin Genet Dev 3: 939–943. 8118220 10.1016/0959-437x(93)90017-j

[16] Wright JM, Bentzen P (1994) Microsatellites: genetic markers for the future. Rev Fish Biol Fish 4: 384–388.

[17] Ferguson A, Taggart JB, Prodöhl PA, McMeel O, Thompson C, et al (1995) The application of molecular markers to the study and conservation of fish populations, with special reference to *Salmo* . J Fish Biol 47: 103–126.

[18] Sunnucks P (2000) Efficient genetic markers for population biology. Trends Ecol Evol 15: 199–203. 10782134 10.1016/s0169-5347(00)01825-5

[19] Schwartz MK, Luikart G, Waples RS (2007) Genetic monitoring as a promising tool for conservation and management. Trends Ecol Evol 22: 25–33. 16962204 10.1016/j.tree.2006.08.009

[20] Tautz D (1989) Hypervariability of simple sequences as a general source for polymorphic DNA markers. Nucleic Acids Res 17: 6463–6471. 2780284 10.1093/nar/17.16.6463 PMC318341

[21] Selkoe KA, Toonen RJ (2006) Microsatellites for ecologists: a practical guide to using and evaluating microsatellite markers. Ecol Lett 9: 615–629. 16643306 10.1111/j.1461-0248.2006.00889.x

[22] Jones AG, Ardren WR (2003) Methods of parentage analysis in natural populations. Mol Ecol 12: 2511–2523. 12969458 10.1046/j.1365-294x.2003.01928.x

[23] Jones GP, Planes S, Thorrold SR (2005) Coral reef fish larvae settle close to home. Curr Biol 15: 1314–1318. 16051176 10.1016/j.cub.2005.06.061

[24] Almany GR, Berumen ML, Thorrold SR, Planes S, Jones GP (2007) Local replenishment of coral reef fish populations in a marine reserve. Science 316: 742–744. 17478720 10.1126/science.1140597

[25] Daly-Engel TS, Grubbs RD, Bowen BW, Toonen RJ (2007) Frequency of multiple paternity in an unexploited tropical population of sandbar sharks (*Carcharhinus plumbeus*). Can J Fish Aquat Sci 64: 198–204.

[26] Planes S, Jones GP, Thorrold SR (2009) Larval dispersal connects fish populations in a network of marine protected areas. Proc Natl Acad Sci U S A 106: 5693–5697. 19307588 10.1073/pnas.0808007106 PMC2659712

[27] Saenz-Agudelo P, Jones GP, Thorrold SR, Planes S (2009) Estimating connectivity in marine populations: an empirical evaluation of assignment tests and parentage analysis under different gene flow scenarios. Mol Ecol 18: 1765–1776. 19243510 10.1111/j.1365-294X.2009.04109.x

[28] Saenz-Agudelo P, Jones GP, Thorrold SR, Planes S (2011) Connectivity dominates larval replenishment in a coastal reef fish metapopulation. Proc Biol Sci 278: 2954–2961. 21325328 10.1098/rspb.2010.2780 PMC3151711

[29] Buston PM, Bogdanowicz SM, Wong A, Harrison RG (2007) Are clownfish groups composed of close relatives? An analysis of microsatellite DNA variation in *Amphiprion percula* . Mol Ecol 16: 3671–3678. 17845439 10.1111/j.1365-294X.2007.03421.x

[30] Wabnitz C, Taylor M, Green E, Razak T (2003) From ocean to aquarium: the global trade in marine ornamental species. UNEP-WCMC. Cambridge, UK.

[31] Fricke H, Fricke S (1977) Monogamy and sex change by aggressive dominance in coral reef fish. Nature 266: 830–832. 865603 10.1038/266830a0

[32] Fautin DG, Allen GR (1994) Anemonefishes and their host sea anemones, Tetra Press, Germany.

[33] Madduppa HH (2012) Self-recruitment in anemonefish and the impact of marine ornamental fishery in Spermonde Archipelago, Indonesia: implications for management and conservation. PhD thesis. University of Bremen, Germany.

[34] Timm J, Kochzius M (2008) Geological history and oceanography of the Indo-Malay Archipelago shape the genetic population structure in the false clown anemonefish (*Amphiprion ocellaris*). Mol Ecol 17: 3999–4014. 19238702 10.1111/j.1365-294x.2008.03881.x

[35] Tomascik T, Mah A, Nontji A, Moosa M (1997) The ecology of Indonesian seas. Part II. Periplus Edition Ltd.

[36] Wyrtki K (1961) Physical oceanography of Southeast Asian waters. NAGA report. The University of California, Scripps Institution of Oceanography. LaJolla, California.

[37] James MK, Armsworth PR, Mason LB, Bode L (2002) The structure of reef fish metapopulations: modelling larval dispersal and retention patterns. Proc Biol Sci 269: 2079–2086. 12396481 10.1098/rspb.2002.2128 PMC1691134

[38] Edinger EN, Jompa J, Limmon GV, Widjatmoko W, Risk MJ (1998) Reef degradation and coral biodiversity in Indonesia: effects of land-based pollution, destructive fishing practices and changes over time. Mar Pollut Bull 36: 617–630.

[39] Edinger EN, Risk MJ (2000) Reef classification by coral morphology predicts coral reef conservation value. Biol Conserv 92: 1–13.

[40] Moyer JT, Nakazono A (1978) Protandrous hermaphroditism in six species of the anemonefish genus *Amphiprion* in Japan. Gyoruigaku Zasshi 25: 101–106.

[41] Wang L, Shi X, Su Y, Meng Z, Lin H (2012) Loss of genetic diversity in the cultured stocks of the Large Yellow Croaker, *Larimichthys crocea*, revealed by microsatellites. Int J Mol Sci 13: 5584–5597. 22754317 10.3390/ijms13055584 PMC3382745

[42] Quenouille B, Bouchenak-Khelladi Y, Hervet C, Planes S (2004) Eleven microsatellite loci for the saddleback clownfish *Amphiprion polymnus* . Mol Ecol Notes 4: 291–293.

[43] Liu SYV, Yu HT, Dai CF (2007) Eight microsatellite loci in Clark’s anemonefish, Amphiprion clarkii. Mol Ecol Notes 7: 1169–1171.

[44] Van-Oosterhout C, Hutchinson WF, Wills DPM, Shipley P (2004) Micro-checker: software for identifying and correcting genotyping errors in microsatellite data. Mol Ecol Notes 4: 535–538.

[45] Nei M (1973) Analysis of gene diversity in subdivided populations. Proc Natl Acad Sci U S A 70: 3321–3323. 4519626 10.1073/pnas.70.12.3321 PMC427228

[46] Botstein D, White RL, Skolnick M, Davis RW (1980) Construction of a genetic linkage map in man using restriction fragment length polymorphisms. Am J Hum Genet 32: 314–331. 6247908 PMC1686077

[47] Marshall TC, Slate J, Kruuk LEB, Pemberton JM (1998) Statistical confidence for likelihood-based paternity inference in natural populations. Mol Ecol 7: 639–655. 9633105 10.1046/j.1365-294x.1998.00374.x

[48] Raymond M, Rousset F (1995) GENEPOP (Version 1.2): Population genetics software for exact tests and ecumenicism. J Hered 86: 248–249.

[49] Rousset F (2008) Genepop’007: a complete re-implementation of the genepop software for Windows and Linux. Mol Ecol Resour 8: 103–106. 21585727 10.1111/j.1471-8286.2007.01931.x

[50] Goudet J (1995) FSTAT (Version 1.2): A computer program to calculate F-statistics. J Hered 86: 485–486.

[51] Weir BS, Cockerham CC (1984) Estimating F-Statistics for the Analysis of Population Structure. Evolution 38 1358–1370. 28563791 10.1111/j.1558-5646.1984.tb05657.x

[52] Glaubitz JC (2004) Convert: A user-friendly program to reformat diploid genotypic data for commonly used population genetic software packages. Mol Ecol Notes 4: 309–310.

[53] Gerber S, Chabrier P, Kremer A (2003) FAMOZ: a software for parentage analysis using dominant, codominant and uniparentally inherited markers. Mol Ecol Notes 3: 479–481.

[54] Meagher TR (1986) Analysis of paternity within anatural population of *Chamaelirium luteum*. 1. Identification of most-likely maleparents. Am Nat 128: 199–215.

[55] Jamieson A, Taylor SCS (1997) Comparisons of three probability formulae for parentage exclusion. Anim Genet 28: 397–400. 9616104 10.1111/j.1365-2052.1997.00186.x

[56] Gerber S, Mariette S, Streiff R, Bodénès C, Kremer A (2000) Comparison of microsatellites and amplified fragment length polymorphism markers for parentage analysis. Mol Ecol 9: 1037–1048. 10964223 10.1046/j.1365-294x.2000.00961.x

[57] Meagher TR, Thompson E (1986) The relationship between single parent and parent pair genetic likelihoods in genealogy reconstruction. Theor Popul Biol 29: 87–106.

[58] Konovalov DA, Manning C, Henshaw MT (2004) Kingroup: a program for pedigree relationship reconstruction and kin group assignments using genetic markers. Mol Ecol Notes 4: 779–782.

[59] Goodnight KF, Queller DC (1999) Computer software for performing likelihood tests of pedigree relationship using genetic markers. Mol Ecol 8: 1231–1234. 10447863 10.1046/j.1365-294x.1999.00664.x

[60] SAS Institute Inc (2012) Base SAS 9.3 Procedure Guide: Statistical Procedures, Second Edition, Cary, NC: SAS Institute Inc.

[61] Erdmann M (1995) The ABC guide to coral reef fisheries in southwest Sulawesi, Indonesia. NAGA The ICLARM quarterly. April: 4−6.

[62] Chozin M (2008) Illegal but common: Life of blast fishermen in the Spermonde Archipelago, South Sulawesi, Indonesia. Ohio University. Ohio.

[63] Caley MJ, Carr MH, Hixon MA, Hughes TP, Jones GP, et al (1996) Recruitment and the local dynamics of open marine populations. Annu Rev Ecol Syst 27: 477–500.

[64] Cowen RK, Lwiza KMM, Sponaugle S, Paris CB, Olson DB (2000) Connectivity of marine populations: open or closed? Science 287: 857–859. 10657300 10.1126/science.287.5454.857

[65] Mora C, Sale PF (2002) Are populations of coral reef fish open or closed? Trends Ecol Evol 17: 422–428.

[66] Carreras-Carbonell J, Macpherson E, Pascual M (2007) High self-recruitment levels in a Mediterranean littoral fish population revealed by microsatellite markers. Mar Biol 151: 719–727.

[67] Jones GP, Milicich MJ, Emslie MJ, Lunow C (1999) Self-recruitment in a coral reef fish population. Nature 402: 802–804.

[68] Miller JA, Shanks AL (2004) Evidence for limited larval dispersal in black rockfish (*Sebastes melanops*): implications for population structure and marine-reserve design. Can J Fish Aquat Sci 61: 1723–1735.

[69] Swearer SE, Shima JS, Hellberg ME, Thorrold SR, Jones GP, et al (2002) Evidence of self-recruitment in demersal marine populations. Bull Mar Sci 70: 251–271.

[70] Cowen RK, Paris CB, Srinivasan A (2006) Scaling of connectivity in marine populations. Science 311: 522–527. 16357224 10.1126/science.1122039

[71] Salas E, Molina-Ureña H, Walter R, Heath D (2010) Local and regional genetic connectivity in a Caribbean coral reef fish. Mar Biol 157: 437–445.

[72] Leis J, Wright K, Johnson R (2007) Behaviour that influences dispersal and connectivity in the small, young larvae of a reef fish. Mar Biol 153: 103–117.

[73] Gerlach G, Atema J, Kingsford MJ, Black KP, Miller-Sims V (2007) Smelling home can prevent dispersal of reef fish larvae. Proc Natl Acad Sci U S A 104: 858–863. 17213323 10.1073/pnas.0606777104 PMC1783404

[74] Atema J, Kingsford MJ, Gerlach G (2002) Larval reef fish could use odour for detection, retention and orientation to reefs. Mar Ecol Prog Ser 241: 151–160.

[75] Polunin NVC, Roberts CM (1993) Greater biomass and value of target coral-reef fishes in two small Caribbean marine reserves. Mar Ecol Prog Ser 100: 167–176.

[76] Birkeland C, Dayton PK (2005) The importance in fishery management of leaving the big ones. Trends Ecol Evol 20: 356–358. 16701393 10.1016/j.tree.2005.03.015

[77] Steneck RS (2006) Staying connected in a turbulent world. Science 311: 480–481. 16439653 10.1126/science.1123541

[78] Ward AJW, Hart PJB (2003) The effects of kin and familiarity on interactions between fish. Fish and Fisheries 4: 348–358.

[79] Wright S (1932) The roles of mutation, inbreeding, crossbreeding and selection in evolution. Proceedings of the 6th International Congress of Genetics, 356−366.

[80] Charlesworth D, Charlesworth B (1987) Inbreeding depression and its evolutionary consequences. Annu Rev Ecol Syst 18: 237–268.

[81] Buston PM, Fauvelot C, Wong MY, Planes S (2009) Genetic relatedness in groups of the humbug damselfish *Dascyllus aruanus*: small, similar-sized individuals may be close kin. Mol Ecol 18: 4707–4715. 19845858 10.1111/j.1365-294X.2009.04383.x

[82] Danilowicz BS, Brown CL (1992) Rearing methods for two damselfish species: *Dascyllus albisella* (Gill) and *D. aruanus* (L.). Aquaculture 106: 141–149.

[83] Ogawa T, Brown CL (2001) Ornamental reef fish aquaculture and collection in Hawaii. Aqua Sci Conserv 3: 151–169.

[84] Johnston G, Kaiser H, Hecht T, Oellermann L (2003) Effect of ration size and feeding frequency on growth, size distribution and survival of juvenile clownfish, *Amphiprion percula* . J Appl Ichthyol 19: 40–43.

[85] Madhu K, Madhu R, Krishnan L, Sasidharan CS, Venugopal KM (2006) Spawning and larval rearing of *Amphiprion ocellaris* under captive condition. Marine Fisheries Information Service, Technical and Extension Series 188: 1–5.

[86] Tucker CS (2000) Off-flavor problems in aquaculture. Reviews in Fisheries Science 8: 45–88.

[87] CITES (2013) CITES (the Convention on International Trade in Endangered Species of Wild Fauna and Flora). Available: http://www.cites.org. Accessed 2013 Sept 24.

[88] Agardy MT (1994) Advances in marine conservation: the role of marine protected areas. Trends Ecol Evol 9: 267–270. 21236850 10.1016/0169-5347(94)90297-6

[89] Bohnsack JA (1998) Application of marine reserves to reef fisheries management. Austral J Ecol 23: 298–304.

[90] Gonzalez A, Lawton JH, Gilbert FS, Blackburn TM, Evans-Freke I (1998) Metapopulation dynamics, abundance, and distribution in a microecosystem. Science 281: 2045–2047. 9748167 10.1126/science.281.5385.2045

[91] Botsford LW, Micheli F, Hastings A (2003) Principles for the design of marine reserves. Ecol Appl 13: 25–31.

[92] Gell FR, Roberts CM (2003) Benefits beyond boundaries: the fishery effects of marine reserves. Trends Ecol Evol 18: 448–455.

[93] Palumbi SR (2004) Marine reserves and ocean neighborhoods: the spatial scale of marine populations and their management. Annu Rev Environ Resour 29: 31–68.

[94] Sale PF, Cowen RK, Danilowicz BS, Jones GP, Kritzer JP, et al.. (2005) Critical science gaps impede use of no-take fishery reserves. Trends Ecol Evol 20:, 74−80. 10.1016/j.tree.2004.11.007 16701346

[95] Thorrold SR, Latkoczy C, Swart PK, Jones CM (2001) Natal homing in a marine fish metapopulation. Science 291: 297–299. 11209078 10.1126/science.291.5502.297

