# Correction: Interspecific, Spatial and Temporal Variability of Self-Recruitment in Anemonefishes

**DOI:** 10.1371/journal.pone.0101396

**Published:** 2014-06-20

**Authors:** The PLOS ONE Staff

There are errors in the Author Contributions section. The correct contributions are: Conceived and designed the experiments: HM MK. Performed the experiments: HM. Analyzed the data: HM JT MK. Contributed reagents/materials/analysis tools: MK. Wrote the paper: HM MK JT.
