# Supplementary material for: Interspecific, Spatial and Temporal Variability of Self-Recruitment in Anemonefishes
Source: PLoS One. 2014 Feb 28;9(2):e90648. doi: 10.1371/journal.pone.0090648 (PMC3938785; doi:10.1371/journal.pone.0090648)
Supplement: Table S1 — Error rates on parentage assignment and gene flow for different populations of parents and juveniles. Bold numbers indicate the LOD thresholds used for parentage analysis in the program Famoz. [Abbreviations: Juvs = Juveniles; BL = Barrang Lompo; S = Samalona; SP = single parent; PP = parent pair]. (DOC) [file pone.0090648.s001.doc]

**Table S1** Error rates on parentage assignment and gene flow for different populations of parents and juveniles. Bold numbers indicate the LOD thresholds used for parentage analysis in the program Famoz. [Abbreviations: Juvs=Juveniles; BL=Barrang Lompo; S=Samalona; SP=single parent; PP=parent pair].

|  |  |  |  | Error rates | |  |  |  |
| --- | --- | --- | --- | --- | --- | --- | --- | --- |
|  | Species | Parents | Juvs | 0 | 0.0001 | 0.001 | 0.01 | 0.1 |
| Threshold (SP) | *A. ocellaris* | BL | BL | 0.6 | 0.7 | 2.9 | **1.9** | 0.2 |
|  | *A. ocellaris* | S | S | 0.9 | 3.8 | 3.7 | **2.5** | 0.7 |
|  | *A. perideraion* | BL | BL | 2.5 | 3.3 | **2.8** | 1.7 | 0.5 |
|  | *A. ocellaris* | S | BL | 0.9 | 3.85 | 3.9 | **2.6** | 0.6 |
|  | *A. ocellaris* | BL | BL | 0.8 | 2.6 | 3.01 | **2.1** | 0.3 |
| Threshold (PP) | *A. ocellaris* | BL | BL | 8.1 | 8.5 | 8.4 | **5.9** | 0.7 |
|  | *A. ocellaris* | S | S | 9.4 | 10.6 | 10.3 | **6.7** | 1.7 |
|  | *A. perideraion* | BL | BL | 9.6 | 9.3 | **7.9** | 4.7 | 0.9 |
|  | *A. ocellaris* | S | BL | 9.5 | 10.75 | 10.7 | **6.8** | 2 |
|  | *A. ocellaris* | BL | BL | 10.4 | 10.2 | 8.4 | **6** | 1.3 |
| Type error I (α) | *A. ocellaris* | BL | BL | 0 | 0.0 | 0.5 | 8.3 | 18.9 |
|  | *A. ocellaris* | S | S | −0.0001 | 1.3 | 4.5 | 27.6 | 145.9 |
|  | *A. perideraion* | BL | BL | 0.19 | 0.3 | 3.4 | 39.4 | 75.2 |
|  | *A. ocellaris* | S | BL | −0.0012 | 1.1 | 4.4 | 30.3 | 107.3 |
|  | *A. ocellaris* | BL | BL | 2.9 | 0.0 | 0.0 | 7.2 | 67.6 |
| Type error II (ß) | *A. ocellaris* | BL | BL | 7.4 | 15.8 | 9.4 | 12.3 | 1.3 |
|  | *A. ocellaris* | S | S | 22.6 | 14.0 | 13.4 | 4.5 | −60.8 |
|  | *A. perideraion* | BL | BL | 4.69 | 6.97 | 10.42 | −2.45 | −74.16 |
|  | *A. ocellaris* | S | BL | 22.8 | 13.7 | 13.2 | 4.9 | −33.9 |
|  | *A. ocellaris* | BL | BL | 3.4 | 6.4 | 9.6 | 10.9 | −63.0 |
| Gene flow | *A. ocellaris* | BL | BL | 0.92 | 0.84 | 0.86 | 0.7 | 0.2 |
|  | *A. ocellaris* | S | S | 0.79 | 0.82 | 0.75 | 0.65 | 0.36 |
|  | *A. perideraion* | BL | BL | 0.72 | 0.88 | 0.75 | 0.56 | 0.13 |
|  | *A. ocellaris* | S | BL | 0.82 | 0.82 | 0.79 | 0.77 | 0.59 |
|  | *A. ocellaris* | BL | BL | 0.97 | 0.97 | 0.97 | 0.94 | 0.23 |
